# Phenotypic and Genotypic Characterization of Antimicrobial Resistance in Streptococci Isolated from Human and Animal Clinical Specimens

**DOI:** 10.1007/s00284-023-03337-6

**Published:** 2023-05-31

**Authors:** Paulina Glajzner, Eligia M. Szewczyk, Magdalena Szemraj

**Affiliations:** Department of Pharmaceutical Microbiology and Microbiological Diagnostics, Medical University of Lodz, Ul. Muszyńskiego 1, 90-001 Łódź, Poland

## Abstract

Recently, the phenomenon of infection of humans as hosts by animal pathogens has been increasing. *Streptococcus* is an example of a genus in which bacteria overcome the species barrier. Therefore, monitoring infections caused by new species of human pathogens is critical to their spread. Seventy-five isolates belonging to streptococcal species that have recently been reported as a cause of human infections with varying frequency, were tested. The aim of the study was to determine the drug resistance profiles of the tested strains, the occurrence of resistance genes and genes encoding the most important streptococcal virulence factors. All tested isolates retained sensitivity to β-lactam antibiotics. Resistance to tetracyclines occurred in 56% of the tested strains. We have detected the MLS_B_ type resistance (cross-resistance to macrolide, lincosamide, and streptogramin B) in 20% of the tested strains. 99% of the strains had tetracycline resistance genes. The *erm* class genes encoding MLS_B_ resistance were present in 47% of strains. Among the strains with MLS_B_ resistance, 92% had the streptokinase gene, 58% the streptolysin O gene and 33% the streptolysin S gene. The most extensive resistance concerned isolates that accumulated the most traits and genes, both resistance genes and virulence genes, increasing their pathogenic potential. Among the tested strains, the gene encoding streptokinase was the most common. The results of the prove that bacteria of the species *S. uberis*, *S. dysgalactiae* and *S. gallolyticus* are characterized by a high pathogenic potential and can pose a significant threat in case of infection of the human body.

## Introduction

In addition to commensals and pathogens of animals and humans, bacteria of the genus *Streptococcus* include species that, although isolated mainly from animals, pose a threat to human health [1]. The presence of these species, especially resistant strains in companion and livestock animals, can be significant, regardless of their transmissibility to humans [2]. Some streptococcal species that are highly pathogenic to humans, such as *S. suis*, are not transmissible between humans [3]. Other species, although less pathogenic, are spreading among humans, representing new human pathogens of increasing importance. Relatively recently, these include *S. dysgalactiae* and *S. gallolyticus*. Other species remain mainly associated with an animal host. So far, single cases of their isolation from human infections, indicate the possibility of adaptation to a new host [4]. Examples of such pathogens are *S. iniae* and *S. uberis* [5–7]. The factor determining the occurrence of diseases in humans caused by these primary animal microbial species is the frequency of contact between humans and the primary animal host. [4].

*S. dysgalactiae* subsp. *equisimilis* (SDSE) constitutes a microbiome not only of animals but also of humans. Human infections caused by this subspecies of *S. dysgalactiae* are infective endocarditis, necrotizing fasciitis, toxic shock syndrome, rheumatic fever or osteoarthritis. Mild diseases include skin infections or tonsillitis. Although more often considered as an animal, *S. dysgalactiae* subsp. *dysgalactiae* (SDSD) is also sometimes isolated from humans [8]. In cattle, isolates of this subspecies have caused mastitis, arthritis, bacteremia and toxic shock syndrome [9]. In humans, they have caused, e.g., infective endocarditis, bacteremia, cellulitis or infection after joint arthroplasty [10]. In *S. dysgalactiae* species, strains with reduced susceptibility to penicillins have occasionally appeared [11].

Strains of *S. gallolyticus*, a species belonging to the SBSEC complex (*Streptococcus bovis*/*Streptococcus equinus* complex), are also increasingly isolated from human infections. They are commonly considered bovine commensals but can cause meningitis and endocarditis in these animals. In humans, *S. gallolyticus* subsp. *pasteurianus* is isolated from urine in urinary tract infections and *S. gallolyticus* subsp. *gallolyticus* from blood in cases of endocarditis [9, 10]. Both of these subspecies inhabiting the human digestive tract also have a clear, widely documented relationship with the formation of cancer of the bile ducts and large intestine [12, 13]. Among the strains of this complex, a worrying increase in antibiotic resistance, including penicillin G and vancomycin, is observed [13].

Strains of *S. uberis* are seldom isolated from humans but very often from bovine milk at various stages of mastitis development [5]. Only a few cases of human infection have been reported so far—skin infection, lower respiratory tract infection, endocarditis, and bacteremia. However, the occurrence of these cases is worrying and suggests the need for monitoring as strains resistant to penicillin and second-line antibiotics have already been isolated [14].

The recommendations of the European Committee for Antimicrobial Susceptibility Testing (EUCAST) provide information that can support standards of treatment for infections. Prepared and updated breakpoints for the assessment of antibiotic resistance to a specific therapeutic group enable the correct selection of the appropriate antimicrobial agent in the case of antibacterial therapy. According to EUCAST recommendations, groups A, B, C, and G streptococcal strains isolated from human clinical cases should be tested first for penicillin susceptibility, followed by macrolides, lincosamides, tetracyclines, and quinolones. For resistant strains, susceptibility to glycopeptides, linezolid, daptomycin, and rifampicin should be determined. The guidelines do not indicate the need for susceptibility testing to other antibiotics. Thus, the possibility of effective treatment with such chemotherapeutics is assumed.

Controlling and monitoring antibiotic resistance is the primary tool in ensuring the long-term effectiveness of antibiotics. The control of antibiotic therapy should cover strains isolated from humans and animals, with particular emphasis on those animal species of bacteria that have demonstrated the ability to cause human disease. Our study focused on showing the phenotypic and genotypic resistance of species rarely isolated from humans. The results will update epidemiological data and improve treatment recommendations for infections caused by primary animal streptococci.

## Materials and Methods

### Tested Strains

Seventy-five streptococcal isolates from human and animal clinical materials were tested. Ten strains of *S. gallolyticus* subsp. *pasteurianus*, one strain *S. gallolyticus* subsp. *gallolyticus* and 34 strains of *S. dysgalactiae* subsp. *equisimilis* (SDSE) were isolated and identified using MALDI TOF MS in clinical laboratories in Lodz and Szczecin, Poland. SDSE strains were isolated from materials such as wounds and skin lesions (*n* = 20), throat (*n* = 5), ear (*n* = 3), vagina (*n* = 3). The three remaining SDSE strains were isolated from peritoneal, nasal and aspirate. Strains of *S. gallolyticus* subsp. *pasteurianus* were isolated from urine (*n* = 4), wounds (*n* = 2), feces and rectum (*n* = 3), one isolate derived from blood. The strain *S. gallolyticus* subsp. *gallolyticus* also derived from blood. Animal isolates: 25 strains of *S. uberis* and five *S. dysgalactiae* subsp. *dysgalactiae* (SDSD) were obtained from clinical samples taken from cows with clinical mastitis.

### Identification of Tested Strains

Strains from animals and *S. gallolyticus* were identified phenotypically (API-STREP) and genetically. The following primers were used for genetic identification: *strep-spp* for the genus *Streptococcus*, *pau* for *S. uberis*, *sdys* for *S. dysgalactiae*, *tanB* for *S. gallolyticus* subsp. *gallolyticus*, *sgpb0680* for *S. gallolyticus* subsp. *pasteurianus* [15–18]. In the case of *S. dysgalactiae* isolated from animals, sequencing of the 16S rRNA genes (the *LPW57* and the *LPW58* gene) were used to identify the subspecies [19]. The DNA sequences determined in this study were deposited in GenBank with accession no. OQ866346-OQ866350.

### Antibiotic Susceptibility Testing Using the Disc Diffusion Method

Antibiotic susceptibility was tested by disc diffusion method according to EUCAST guidelines for group A, B, C, and G streptococci and Viridans group [20]. The following antibiotics were used: penicillin, norfloxacin, tetracycline, tigecycline, erythromycin, clindamycin, chloramphenicol, rifampicin, sulfamethoxazole with trimethoprim, vancomycin, and linezolid. For strains from the Viridans group, ampicillin, cefepime and cefuroxime were additionally used.

### Detection of Antibiotic Resistance and Virulence Genes

DNA of the tested strains was isolated using the Genomic Micro AX Bacteria + Gravity kit according to the manufacturer's instructions (A&A Biotechnology, Poland). Penicillin resistance genes (*pbp2b*), tetracyclines (*tetK, tetL, tetM, tetO, tetS*), macrolides and lincosamides (*ermA, ermB, ermC*, *ermTR, mefA, lnuA* and *lnuD*), fluoroquinolones (*parC, gyrA*), and aminoglycosides (*aad6, aphA*) were screened by PCR using the previously described primers and parameters [21–25]. The *ska, slo, sagA* virulence genes encoding streptokinase, streptolysin O and streptolysin S were searched for selected strains [9]. Reaction products were electrophoretically separated on 1% agarose gels containing Midori Green DNA dye (Nippon Genetics Europe, Germany) at 70 V for 1 h.

## Results and Discussion

The tested isolates came from clinical materials of human and animal infections and were collected within the last two years. In susceptibility testing of all isolates, we followed EUCAST recommendations for the treatment of infections in humans. The number of clinically resistant isolates to the indicated antibiotics within the tested species is presented in Table 1.

**Table 1:** Antimicrobial resistance profile of streptococci isolated from human and animal clinical specimens

Antibiotic class	Number (%) of resistant *Streptococcus* spp.			
	*S. uberis* (*n* = 25)	*S. dysgalactiae* subsp. *dysgalactiae* (*n* = 5)	*S. dysgalactiae* subsp. *equisimilis* (*n* = 34)	S. *gallolyticus* (*n* = 11)
Penicillins (Benzylpenicillin)	0 (0)	0 (0)	0 (0)	0 (0)
Tetracyclines (Tetracycline)	5 (20)	5 (100)	22 (65)	10 (91)^a^
Fluoroquinolones (Norfloxacin)	0 (0)	0 (0)	0 (0)	7 (0)^b^
Macrolides (Erythromycin)	2 (8)	1 (20)	9 (26)	3 (27)
Lincosamides (Clindamycin)	2 (8)	1 (20)	9 (26)	4 (36)
Ryfamycin (Rifampicin)	1 (4)	0 (0)	1 (3)	0 (0)

*n* number of tested strains

^a^No EUCAST recommendations, result based on discs sensitivity method for groups A, B, C and G streptococci (inhibition zone: 0–19 mm)

^b^No EUCAST recommendations, result based on discs sensitivity method for groups A, B, C, and G streptococci, considered only in cases of uncomplicated UTI; (inhibition zone: 0-11 mm)

For streptococcal infections, penicillins are usually the first-line therapy. Many studies now indicate the appearance of reduced susceptibility to this group of drugs caused by the occurrence of mutations in penicillin-binding proteins. This mechanism has been observed, e.g., among strains of *S. pneumoniae*, *S. agalactiae*, *S. suis*, and *S. uberis* [26]. None of the strains we tested developed resistance to penicillin, which is the first-line antibiotics. The penicillin resistance gene *pbp2b* was also not detected.

Among the tested strains, phenotypic resistance and the presence of tetracycline resistance genes were common. Tetracycline-resistant isolates contained the *tetM* and *tetK* genes, both of which, in addition to the t*etL*, *tetS*, and *tetO* genes, were also found in susceptible isolates. This proves the lack of expression of genes encoding resistance to tetracyclines among some strains. The most common was the *tetM* gene. This gene was present in 70 tested strains (93%). The prevalence of its occurrence is associated with frequent transmission within the genus *Streptococcus* [21]. The presence of the *tetM* and *tetO* genes pair and the *tetK* and *tetM* genes pair were most frequently observed among all tested strains. The presence of the *tetO* gene has been found certainly more often in the strains isolated from humans, regardless of the species. This gene appeared in 31 of the 45 strains (69%) isolated from humans and only three of the 30 strains (10%) isolated from animals. In recent years, the *tetO* gene rarely appears in strains isolated from animals [27]. The *tetM*, *tetO* and *tetS* genes encoding resistance by protecting bacterial ribosomes were more frequent in our strains than the *tetK* and *tetL* genes encoding the efflux pump [22]. The resistance to tetracyclines for *S. uberis* reported in the literature is up to 40%, and for bovine strains of *S. dysgalactiae*—up to 85%. Resistance to this group of drugs in our studies concerned half of the isolates, 32 strains *S. uberis* and *S. dysgalactiae*, and could result from the widespread use of tetracyclines in animal breeding and treatment. Tetracyclines were also one of the main classes of antibiotics used in veterinary medicine in Poland, and until 2006 they were used in subtherapeutic doses as an additive to animal feed [22, 28].

Streptococci are also highly sensitive to fluoroquinolones. All tested strains of *S. uberis* and *S. dysgalactiae* were susceptible to fluoroquinolones. The sought genes encoding resistance to fluoroquinolones were the *parC* gene and the *gyrA* gene. Among them, however, the resistance *parC* gene was abundant. The *parC* gene was found in 39% of the tested strains. The *gyrA* gene was present in only three strains. Genes of resistance to this group of antibiotics occurred in 30 tested strains. The genes *parC* and *gyrA* are responsible for mutations in topoisomerase IV and gyrase, respectively. In gram-positive bacteria, the most common mutations concern topoisomerase IV and the transfer of the *parC* gene. Among strains of *S. pneumoniae* and *S.* *suis*, mutations within the gyrase gene were often detected, although resistance to fluoroquinolones was relatively rare [29, 30].

**Fig. 1:**
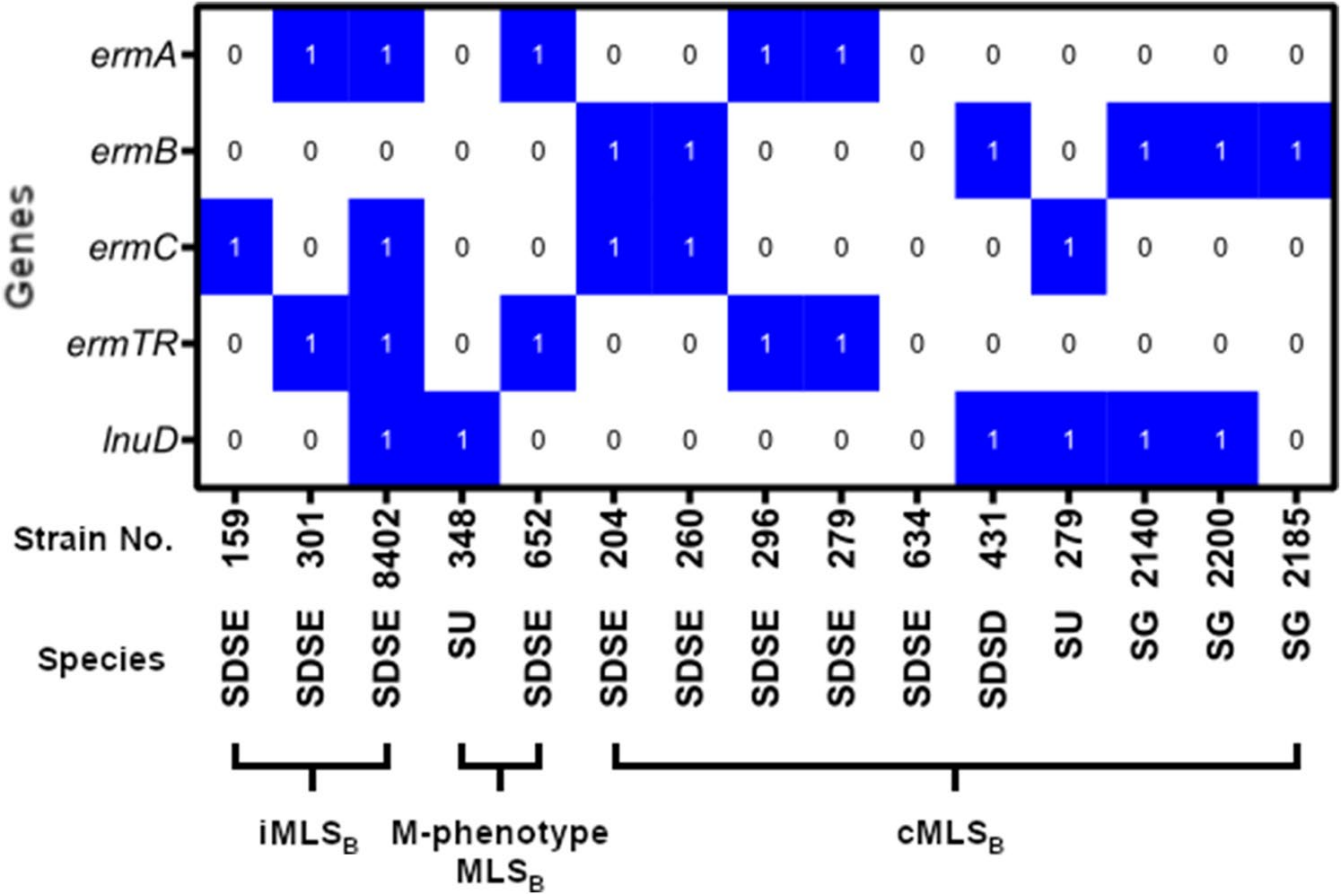
Distribution of the *erm* class and *lnu* resistance genes in strains with different MLS_B_ resistance phenotypes. *SDSE S. dysgalactiae* subsp. *equisimilis*, *SU S. uberis*, *SDSD S. dysgalactiae* subsp. *dysgalactiae*, *SG S. gallolyticus*, *1 *present gene, *0 *absent gene

For resistance to macrolides, lincosamides and streptogramin B, both constitutive (cMLS_B_) and inductive (iMLS_B_) types were observed among the 15 resistant strains. cMLS_B_ type was detected in one *S. uberis* isolate, five SDSE isolates and three *S. gallolyticus*, while iMLS_B_ type in three SDSE isolates. Two isolates (one *S. uberis* and one SDSE) had an M-phenotype, retaining susceptibility to lincosamides, and one SDSD was resistant to macrolides and lincosamides. Three isolates of the tested strains were resistant only to lincosamides. The relationship between MLS_B_ resistance mechanisms and the presence of resistance genes is shown in Fig. 1. MLS_B_ resistance was most often associated with the presence of at least two genes from the *erm* gene class: *ermA* + *ermTR* and *ermB* + *ermC*. Among *S. gallolyticus* strains, MLS_B_ resistance was related to the presence of the *ermB* gene. It was noted that only macrolides could induce resistance to MLS_B_, which was most often associated with the presence of the *ermB* gene in each of the studied streptococcal species [2, 21].

All *erm* genes responsible for encoding methylase were found in the tested strains. This enzyme reduces the binding of antibiotics—macrolides, lincosamides and streptogramin B—to the target site [22]. The most common genes were *ermB* and *ermC*. Genes encoding resistance to MLS_B_ were much more common among strains of *S. dysgalactiae* subsp. *equisimilis* and *S. gallolyticus* (*n* = 29) isolated from human infections than strains isolated from animals. The lincosamide *lnuD* resistance gene was as frequent as the *erm* genes. It is responsible for encoding nucleotidyltransferase. The presence of the *lnuD* gene in *S. agalactiae* and *S. uberis* has only recently been demonstrated. These genes are widely distributed among environmental bacteria [21]. Interestingly, of the 57 macrolide- and lincosamide-sensitive isolates, only 15 lacked any of the genes associated with this resistance. 42 strains sensitive to macrolides and lincosamides are carriers of the so-called silent genes that are not expressed, in this case not leading to phenotypic resistance. However, under appropriate conditions, e.g., in vivo, they can be expressed and lead to therapeutic failures [31].

Two rifampicin-resistant isolates were also resistant to other antibiotics. The SDSE isolate was resistant to tetracycline and erythromycin (iMLS_B_ mechanism); *S. uberis* isolate presented an M-phenotype of resistance to macrolides.

In the treatment of streptococcal infections, aminoglycosides are used in combination with penicillins. Streptococci are naturally poorly sensitive to aminoglycosides due to limited penetration through their cell wall. Strains resistant to gentamicin, streptomycin and kanamycin have been described [21]. We found the aminoglycoside resistance genes *aad6* and *aphA* responsible for the enzymatic inactivation of these antibiotics in 30% of the strains we tested.

**Fig. 2:**
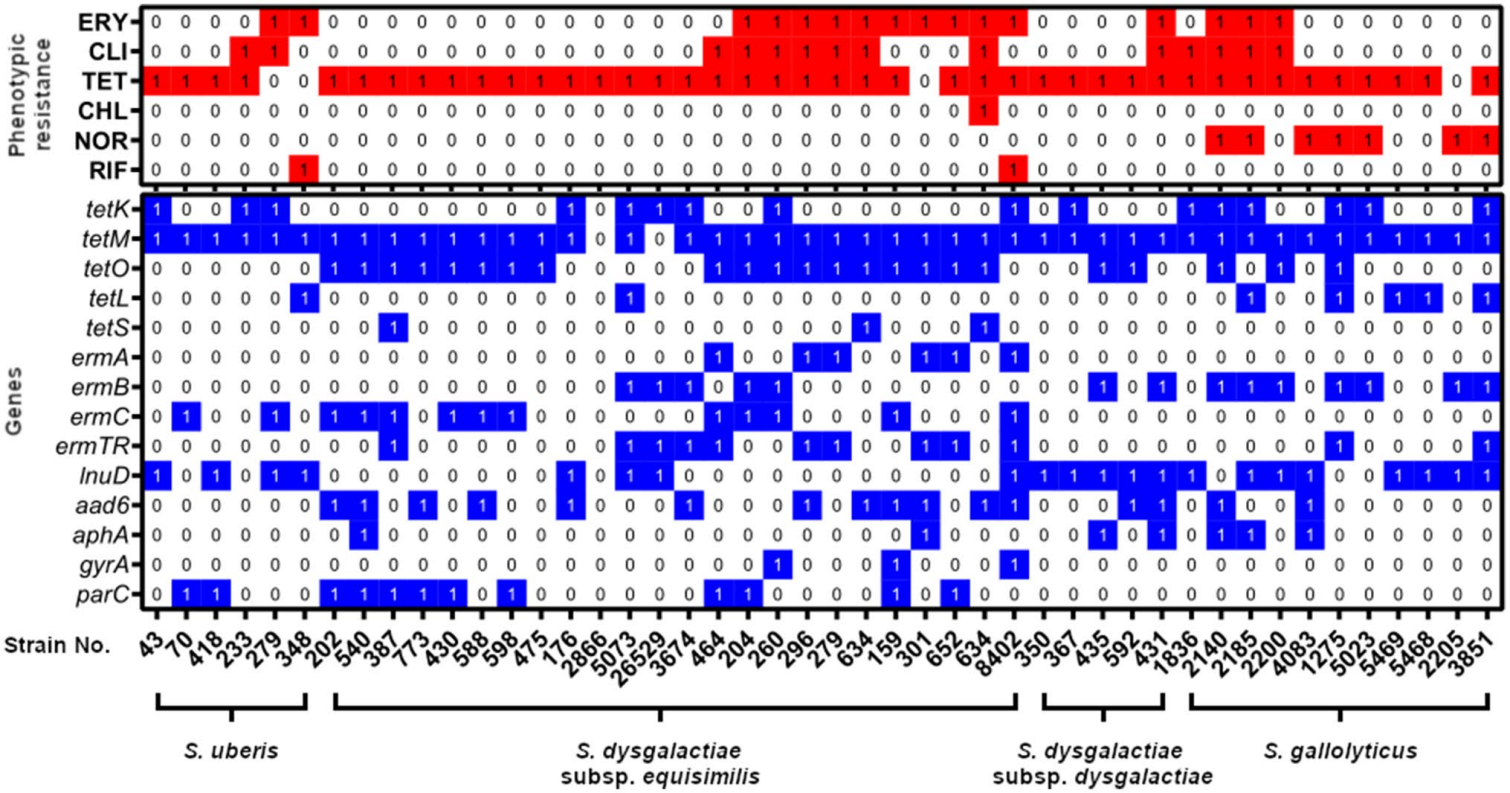
Occurrence of antibiotic resistance genes among strains resistant to tetracyclines, macrolides, lincosamides, chloramphenicol, norfloxacin and rifampicin. *ERY *erythromycin, *CLI *clindamycin, *TET *tetracycline, *CHL *chloramphenicol, *NOR *norfloxacin, *RIF *rifampicin

The results of the genetic studies of strains resistant to all antibiotics tested in this study are presented in Fig. 2.

The presence of the sought genes responsible for resistance in all considered isolates did not always mean a phenotypic manifestation of this trait. This phenomenon was also observed in earlier studies [2, 21, 32]. Analysis of this phenotypic resistance suggests that other genes that we did not look for were also responsible for it.

Among the species evaluated, the highest resistance was found in *Streptococcus dysgalactiae*. Identification of subspecies of this species has been the subject of research and discussion for years [8]. It was assumed that β-hemolytic strains isolated from humans are SDSE and α-hemolytic strains of animal origin are SDSD. The reliable subspecies assignment must be based on sequencing as both can infect animals and humans, and the hemolytic trait is not sufficiently discriminatory. In our studies, all strains from human infections belonged to the subspecies *S. dysgalactiae* subsp. *equisimilis*, as was demonstrated by sequencing. Infections in humans, however, can also be caused by SDSD [8]. The tested strains isolated from animals came from cattle and were assigned to the SDSD subspecies. However, strains infecting companion animals sometimes have been identified as SDSE [33]. Our studies have shown that the resistance of this species to drugs used in human therapy applies to both subspecies. Treatment of infections caused by them is undoubtedly a therapeutic challenge, especially in people allergic to penicillins.

Both subspecies of *S. gallolyticus* have been isolated from infection in humans only recently, but with alarming frequency. In addition, biofilm formation by these bacteria and migration have been implicated as a mechanism for creating metastatic niches that provide an environment suitable for tumour cell proliferation and tumorigenesis. This phenomenon is alarming in cases of colonization of the large intestine by these streptococci [34]. Infections caused by *S. gallolyticus* subsp. *pasteurianus* (SGP) are primarily meningitis and bacteremia that have been described in both humans and animals. In animals, a high mortality rate is observed. *S. gallolyticus* subsp. *gallolyticus* (SGG) are responsible for endocarditis and bacteremia. Cases of osteomyelitis and meningitis have also been reported. SGG is recognized as the leading pathogen in this type of infection in many countries [35–37]. Among the streptococci of the SBSEC complex, a high degree of resistance to antibiotics is indicated. Resistance to macrolides, lincosamides and tetracyclines has been described [38]. SGP strains isolated from meningitis with reduced susceptibility to penicillin have also been found. Meanwhile, in the treatment of these infections, a combination of β-lactam antibiotics and aminoglycosides is often used [39].

The isolates of the species we studied accumulate many resistance genes. The presence of these genes was common in both clinically resistant and susceptible isolates. These pathogens pose a threat because of their drug resistance and as a reservoir of resistance genes. As a result of horizontal gene transfer (HGT), these strains acquire resistance and many genes useful for the colonization of new hosts, including invasins interfering with the human immune system. Their presence has already been demonstrated in strains of various species of streptococci [9]. An important factor involved in the development of the disease is streptokinase, responsible for the conversion of plasminogen to plasmin, which enhances the spread of streptococci by destroying fibrin clots. Streptolysins O and S can disrupt cytoplasmic membranes, including epithelial cells, erythrocytes, leukocytes, platelets and macrophages. Streptokinase and streptolysin O enable bacteria to survive, replicate and persist in keratinocytes, epithelial cells and macrophages [1].

Of the 12 streptococcal tested strains resistant to tetracycline and MLS_B_, all had the streptokinase gene, and half had either the streptolysin O or streptolysin S gene. This demonstrates their pathogenic potential if an infection develops. A summary of the results showing the resistance profiles and occurrence of these invading agents in these strains is presented in Table 2.

**Table 2:** Characteristics of selected resistant isolates involved in the research

Species	Strain number	Isolation site	Resistance phenotype	Resistance genes	Pathogenicity genes
SDSE	159	Throat	T, iMLS_B_	*tetM, tetO, ermC, aad6, parC, gyrA*	*ska, slo, sagA*
	204	Ear	T, cMLS_B_	*tetM, tetO, ermB, ermC, parC*	*ska*
	260	Ear	T, cMLS_B_	*tetK, tetM, tetO, ermB, ermC, gyrA*	*ska*
	296	Wound	T, cMLS_B_	*tetM, tetO, ermA, ermTR, aad6*	*ska, slo*
	279	Wound	T, cMLS_B_	*tetM, tetO, ermA, ermTR,*	*ska, slo*
	652	Wound	T, M-Phenotype MLS_B_	*tetM, tetO, ermA, ermTR, parC*	*ska, slo*
	634	Wound	T, cMLS_B_, CH	*tetS, tetM, tetO, aad6*	*ska, slo, sagA*
	8402	Aspirate	T, iMLS_B_, R	*tetK, tetM, ermA, ermC, ermTR, lnuD, aad6, gyrA*	*ska, slo, sagA*
SDSD	431	Cow milk	T, cMLS_B_	*tetM, ermB, lnuD, aad6, aphA*	*ska, slo*
SGP	2200	Feces	T, cMLS_B_	*tetM, tetO, ermB, lnuD*	*ska*
	2185	Urine	T, cMLS_B_	*tetK, tetL, tetM, ermB, lnuD, aphA*	*ska*
	2140	Wound	T, cMLS_B_	*tetK, tetM, tetO, ermB, aad6, aphA*	*slo, sagA*

T-tetracycline, *iMLS*_*B*_ MLS_B_ inductive mechanism, *cMLS*_*B*_ MLS_B_ constitutive mechanism, *CH* chloramphenicol, *R* rifampicin, *SDSD S. dysgalactiae* subsp. *dysgalactiae*, *SDSE S. dysgalactiae* subsp. *equisimilis*, *SGP S. gallolyticus* subsp. *pasteurianus*

## Conclusion

The adaptation of animal species to the human body seems to be a natural phenomenon, progressing over time. However, in recent years this process has accelerated significantly. Within the species of *S. dysgalactiae*, *S. gallolyticus* and *S. uberis* that threaten human health, disturbing changes have been observed at the genotypic and phenotypic levels over the years. Identifying species on the way to becoming human pathogens is essential for rational antibiotic therapy and preventing dangerous reservoirs of resistance genes.

We hypothesize that controlling and monitoring antibiotic resistance could be a key tool in ensuring the long-term effectiveness of antibiotics. The proposed monitoring should include strains isolated from humans and animals, in particular strains of zoonotic species that have recently caused human infections. As we have shown, zoonotic streptococci possess a set of resistance genes but also virulence traits, the expression of which can contribute to the development of infection and affect the treatment options for the new host—humans. Among streptococci, the gene encoding the streptokinase and streptolysin genes is common. In addition, resistance to tetracyclines as well as macrolides and lincosamides is common. Strains of these species also possess multiple resistance genes not only to these two classes but also to aminoglycosides and fluoroquinolones.

## Data Availability

All data are available with corresponding author.
